# Food Sustainability Among Dietitians in Saudi Arabia: Assessment of Knowledge, Attitudes, Practices, and Food Consumption

**DOI:** 10.3390/nu18182966

**Published:** 2026-09-10

**Authors:** Abeer A. Aljahdali

**Affiliations:** 1 Department of Clinical Nutrition, King Abdulaziz University, Jeddah 21589, Saudi Arabia; aaoaljahdali1@kau.edu.sa; 2 Department of Nutritional Sciences, University of Michigan, Ann Arbor, MI 48109, USA

**Keywords:** sustainable diets, dietitians, environmental sustainability, nutrition education, Saudi Arabia, knowledge, attitudes, practices, food sustainability, food consumption

## Abstract

**Background**: Dietitians play a crucial role in promoting sustainable dietary patterns aimed at maintaining both human and planetary health. Several studies have investigated knowledge, attitudes, and practices (KAPs) regarding food sustainability among dietitians; nevertheless, evidence from the Middle East remains scarce, with no studies conducted in Saudi Arabia. **Objective**: This study aimed to assess KAPs regarding sustainable dietary patterns and dietary adherence among dietitians in Saudi Arabia. **Methods**: An online structured questionnaire was distributed to collect self-reported information on food sustainability, including knowledge and perceived understanding, perceived importance of key concepts, behaviors, and food consumption. A total of 221 dietitians completed the survey. **Results**: Dietitians reported greater engagement with health-oriented and practical sustainability behaviors, particularly food waste reduction, fruit and vegetable consumption, and affordability-related choices. Also, gaps in dietitians’ knowledge and familiarity were identified regarding environmental metrics of food sustainability concepts. Dietitians reported that food consumption was characterized by frequent consumption of animal-based foods, moderate consumption of fruits, vegetables, legumes, and nuts, and low adoption of plant-based alternatives. **Conclusions**: Dietitians in Saudi Arabia generally exhibited favorable attitudes toward food sustainability, with a gap identified in factual knowledge despite an overestimation of the perceived knowledge and practices pertaining to environmental sustainability, and these gaps did not consistently translate into sustainable food consumption behaviors. These findings highlight the importance of incorporating food sustainability, including environmentally related concepts, into nutrition education and professional development as a potential strategy to bridge the gap between knowledge and application in dietetic practices.

## 1. Introduction

Food systems are major determinants of human and planetary health. Dietary patterns are major lifestyle factors associated with human health and well-being, and strong evidence has shown that poor dietary quality is associated with mortality and morbidity from chronic diseases globally [1,2], emphasizing the importance of higher dietary quality in mitigating the burden of non-communicable diseases. Furthermore, food production and consumption have been substantially associated with planetary health. Food systems result in negative consequences on the environment; food production is associated with soil degradation, water depletion, and greenhouse gas emissions [3], and it has been recognized as the third-highest contributor to overall greenhouse gas emissions [4,5]. Animal food production was responsible for 57% of diet-related greenhouse gas emissions [4]. It has been estimated that food consumption will be responsible for adding approximately 1  °C to global warming by 2100 [6], which would surpass the Paris Agreement 2015’s goal of limiting the total increase by less than 2 °C [7]. It is worth noting the direct association between food systems and planetary health; food systems contribute to climate change, and climate change will in turn challenge the food system. Thus, researchers have concluded that climate change will raise the risk of food insecurity, malnutrition, and diet-related mortality [8].

Therefore, sustainable dietary patterns have been promoted as a solution to mutually mitigate climate change and other negative planetary consequences while supporting human health and well-being [7,9,10]. Sustainable diets focus on multiple dimensions of economy and affordability, social and cultural acceptability, environmental protection, and nutrition and health [11]. In 2025, the EAT–Lancet sustainable reference diet emphasized that approximately half of the plate should be composed of fruits and vegetables, while the remaining part should prioritize whole grains, legumes, nuts, unsaturated plant oils, and modest amounts of animal-source foods. This diet represents a predominantly plant-based or flexitarian dietary pattern [12]. Previous studies have shown that sustainable dietary patterns are associated with reduced risks of all-cause mortality, cardiovascular disease, type 2 diabetes, metabolic dysfunction-associated steatotic liver disease, and asthma, while also reducing greenhouse gas emissions [13]. Therefore, it is important to promote the adoption of a healthy and sustainable diet and food culture to achieve the long-term solutions planned for human and environmental health.

Dietitians have made substantial and pivotal contributions in advocating and promoting sustainable food systems for human and planetary health given their roles and responsibilities in translating scientifically evidenced knowledge into dietary recommendations, which are disseminated through their dietetic practices, institutional food services, community and public health programs and initiatives, or nutrition and food-related policies [14,15,16]. International professional standards recognize the importance of dietitians in shifting toward more sustainable food systems and the demand for including sustainability in dietetic competencies. In 2020, the Academy of Nutrition and Dietetics published a revised Standards of Professional Performance (SOPP) that describes dietetic sustainability-specific competencies across different levels of practice (i.e., competent, proficient, and expert) to help in enhancing “sustainable, resilient, and healthy food and water systems” [16]. By the same token, the Food Systems and Environmental Sustainability Role Statement has been released in Australia [17], and sustainability has been integrated into the National Competency Standards for Dietitians in Australia [18]. Furthermore, Barbour et al. published a position statement for Australian dietitians to intensify the importance of dietitians’ responsibilities and enhance workforce capacities for supporting a transformation in food systems and promoting sustainable dietary practice transformations benefiting human and planetary health [19].

In several studies assessing the importance of food sustainability among nutrition and dietetics professionals, the majority of them believed that promoting sustainable food and supporting sustainable food systems are part of their responsibilities [20,21,22,23]. Similarly, Australian undergraduate nutrition and dietetic students reported their understanding that sustainability is important to their future career in nutrition and dietetics [24]. In fact, nutrition and dietetics professionals across the U.K, Canada, Australia, and the U.S reported actual involvement in sustainable food systems across socioecological levels, i.e., at the individual, community, institutional, and policy-related levels [25,26,27]. Therefore, attention has been given to exploring dietitians’ knowledge, attitudes, and practices (KAPs) about sustainable diets. Previously, studies on food sustainability among dietitians have been conducted in Ireland [28], Australia [24], Canada [15,25], Europe, North America, Oceania [26,27,29], Europe [22], and Turkey [30].

Despite the growing amount of research about food sustainability conducted among dietitians, to the best of the author’s knowledge, no study has been conducted on nutrition and dietetics professionals from the Middle East. This research gap is of great importance because international findings cannot be generalized to Middle Eastern countries due to differences in nutrition curricula and dietetic practices. Meanwhile, there is a substantial burden of non-communicable diseases in Middle Eastern countries, accounting for two-thirds of all deaths in the region [31]. Investigating the status of knowledge, attitudes, and practices (KAPs) surrounding food consumption and food sustainability will result in assessing the needs and guiding the restructuring of nutrition curricula and professional development initiatives. Therefore, the present study aims to assess KAPs and food consumption related to food sustainability among dietitians in Saudi Arabia.

## 2. Materials and Methods

### 2.1. Study Design and Participants

This cross-sectional web-based survey was conducted between 28 December 2025 and 14 April 2026 to investigate the KAPs of registered dietitians in Saudi Arabia toward food sustainability and their adherence to a study-specific sustainable food consumption index. Due to the exploratory nature of this study, no a priori sample-size calculation was conducted, so the final sample size for the study represents the number of completed surveys from the eligible population during the data collection period. Convenience and snowball sampling methods were used to collect self-reported data. Survey dissemination was conducted (1) through multiple social media platforms (X™, WhatsApp™, and Instagram™), where the questionnaire was uploaded via a Google Form, and (2) through a collaboration with the Saudi Commission for Health Specialties, which shared the survey with the registered dietitians in their database, where the questionnaire was uploaded via Qualtrics. Also, participants were asked to disseminate the survey to their colleagues. Initially, there were 416 responses collected: 11 responses through the Google Form survey, which all were complete responses, and 405 responses through Qualtrics, of which 192 responses were excluded due to incompletion, and three participants did not agree to enroll in the study; therefore, only 210 responses from Qualtrics were used, and as a result, the final sample size was 221. The eligibility criteria included being a certified dietitian by the Saudi Commission for Health Specialties (SCFHS). The study was approved by the Research Ethics Committee of the King Abdulaziz University Hospital Biomedical Ethics Unit (IRB No. 246-25) and registered with the National Committee of Bioethics (NCBE; Registration No. HA-02-J-008). Ethical approval was granted on 17 June 2025. The study was conducted according to the ethical principles of the Declaration of Helsinki. Electronically informed consent was obtained from all participants prior to their inclusion in the study. Participation was voluntary, and participants were informed of their right to withdraw at any time. Confidentiality and anonymity were maintained throughout the data collection process.

### 2.2. Study Questionnaire

The questionnaire used in the study was previously used in similar research on food sustainability in the United Arab Emirates (UAE), and permission was granted by the authors to use their questionnaire [32]. The available version of the Hilary et al. questionnaire was in English [32]; the questionnaire was translated into Arabic by two bilingual experts holding Ph.D. degrees in nutrition. The Arabic questionnaire was then sent to seven experts holding Ph.D. degrees in nutrition as part of face and content validity, with a goal of examining the clarity, relevance, and appropriateness of the items. Nevertheless, a formal psychometric validation, including test–retest reliability, was not performed for the Arabic version used in this study. For all items in the current questionnaire, the key answers and scoring criteria were followed as described in the original questionnaires [32,33,34]. The questionnaire consisted of four sections:

• Demographic questions, which focused on sex, age, marital status, region, education level, and monthly household income.
• Work experience profile questions, which included questions on the type of work institute, years of experience, number of clients seen weekly, and a few questions about the knowledge and practices of food sustainability.
• KAP and dietary practices questions adapted from a previous publication [32].
  ⚬ For the perceived knowledge domain, there were seven statements for common sustainability terms, with the responses “No, I have not heard the term,” “I have heard the term, but I do not know what it means,” “I have a vague understanding of the term,” “I have heard the term and know what it means,” “I have a clear understanding of the term” coded as 0, 1, 2, 3, and 4, respectively. Also, there was a question about the similarity between healthy and sustainable diets, with the responses “strongly agree”, “agree”, “neither agree nor disagree”, “disagree”, and “strongly disagree” coded as 0, 1, 2, 3, and 4, respectively. The score ranges from 0 to 32, where higher scores reflect a high perceived knowledge about sustainable diets.
  ⚬ For the attitude domain within the administered version of the questionnaire, there were 17 statements about the importance of certain behaviors toward sustainable diets, with the responses “not at all important”, “slightly important”, “moderately important”, “very important”, and “extremely important” coded as 0, 1, 2, 3, and 4, respectively. The score ranges from 0 to 68, where higher scores reflect highly favorable attitudes toward a sustainable diet.
  ⚬ For the practice domains, 15 statements assessed the degree of practicing sustainable behaviors, with the responses “I’m not interested in doing this at the moment,” “I’m thinking about this, but I need more information,” “I would like to do this, but other things are stopping me,” “I have started to do this some of the time,” and “I’m doing this confidently most of the time”. Answers were coded as 0, 1, 2, 3, and 4, respectively. Another statement asked respondents to report their degree of willingness, including “To what extent are you willing to pay more money for food and drinks that are produced sustainably?”, answer options were “unwilling”, “somewhat unwilling”, “undecided”, “somewhat willing”, and “willing”, with answers coded as 0, 1, 2, 3, and 4, respectively. The sum of the 16 scores was calculated, and the score ranges from 0 to 64, with higher scores reflecting increased practices of sustainable behaviors.
  ⚬ For the food consumption domain, adherence to the food sustainability study-specific index of food consumption was assessed by inquiring about the self-reported frequency of consumption per week of 12 food groups: (1) dairy products (e.g., milk, cheese, yogurt, labneh), (2) white meat (e.g., chicken, turkey), (3) nuts and seeds, (4) red meat (e.g., beef, lamb, camel meat), (5) eggs, (6) processed meat (e.g., sausages, hot dogs, nuggets, beef bacon), (7) beans, lentils, chickpeas, and fava beans, (8) fish, (9) meat alternatives (e.g., tofu, soya products, plant-based sausages/burgers), (10) non-dairy milk (e.g., soy, almond, coconut, oat), (11) fruits, and (12) vegetables (not including legumes and pulses). Available responses for weekly frequency of consumption were 0, 1–2, 3–4, 5–6, and ≥7 per week. The answers were coded as 0, 1, 2, 3, and 4, with higher scores given for higher frequency of consumption for the food groups, except for (1) white meat, (2) red meat, and (3) processed meat, where higher scores were given for lower consumption. The sum of the scores from the 12 food groups was calculated, and the score ranges from 0 to 48, where higher scores reflect a greater adherence to the study-specific index of food consumption related to food sustainability and vice versa.
  ⚬ Three questions were added to examine the factual knowledge: (1) food item where its production is associated with the highest carbon dioxide emissions (answer options: vegetables, chickens, fish, and beef (correct answer)); (2) food item whose growth or raising requires the most water for a typical serving (answer options: grains, beef (correct answer), melons, and lettuce); and (3) the most sustainable diet (answer options: Lacto-ovo-vegetarian with a moderate amount of animal protein in the form of red meat, Lacto-ovo-vegetarian with a small amount of animal protein in the form of fish and/or chicken (correct answer), Lacto-ovo-vegetarian without any meat, and vegan (no animal protein)). A point was given for each correct answer and 0 for incorrect answers [33]. Furthermore, respondents were asked to identify the impact of foods on planetary sustainability: the food items were plant-based food, red meat, processed meat, milk, and processed foods, and the answer options were large impact, medium impact, low impact, and “I don’t know.” For plant-based food, the correct answer was “low impact”, and for the rest of the items, the correct answer was “high impact.” Three points were given for each correct answer, two points were given for “medium impact”, one point was given for an incorrect answer, and 0 was given for “I don’t know.” [34]. The sum of the eight questions was calculated, and the score ranges from 0 to 18, where higher scores reflect greater factual knowledge about food sustainability.

### 2.3. Statistical Analysis

The statistical analyses were performed using the Statistical Analysis System (SAS) 9.4 TS1M7 (SAS Institute Inc., Cary, NC, USA). Descriptive analysis was run for the collected data and is presented as mean ± standard deviation (SD) or median and interquartile range (IQR) for normally distributed continuous variables and non-normally distributed continuous variables, or frequencies and percentages for categorical variables. The Kolmogorov–Smirnov test was used to assess normality, and a *p*-value less than 0.05 indicated a violation of the normality assumption.

Because the study’s questionnaire had two types of knowledge-related questions (i.e., perceived knowledge and factual knowledge), the two scores were combined to reflect an overall knowledge score. Before doing so, the two scores were standardized to enhance the compatibility of the scores and their distribution. The method of standardization used was z-score transformation, where each score had a mean of 0 and a standard deviation of 1. After that, an overall knowledge score was created as an arithmetic average of the two standardized knowledge scores. Nevertheless, as supplementary analysis, the results from perceived knowledge and factual knowledge are presented, as these represent distinct constructs (Supplementary Table S1).

Correlations between each of the KAPs and sustainable dietary practices were performed using Pearson or Spearman correlation coefficients based on the normal distribution of the total score of each component. Classification and interpretation of Pearson’s and Spearman’s correlations are as follows: 0, 0.1–0.2, 0.3–0.5, 0.6–0.7, 0.8–0.9, and 1 represent none, poor, fair, moderate, very strong, and perfect, respectively [35]. Cronbach’s alpha was used to examine internal consistency, and raw Cronbach’s alpha values were reported for perceived knowledge, attitudes, practices, and food consumption. The standardized Cronbach’s alpha was reported for factual knowledge given the variability in scoring ranges. The classifications and interpretation of Cronbach’s alpha are as follows: 0, 0.1–0.2, 0.3–0.5, 0.6–0.7, 0.8–0.9, and 1 represent none, poor, fair, moderate, very strong, and perfect, respectively, while >0.90, 0.80–0.90, 0.70–0.80, 0.60–0.70, 0.50–0.60, and <0.50 represent excellent, good, acceptable, questionable, poor, and unacceptable, respectively [36,37]. All statistical tests were two-tailed, and a *p*-value < 0.05 was considered statistically significant.

## 3. Results

Table 1 shows the descriptive characteristics of the 221 dietitians enrolled in the current study. Female dietitians accounted for 80% of the sample. Regional distribution showed that the highest representation was from the Western and Central regions (33% each), while the Eastern and Northern regions represented 10% each. The predominant level of education was a bachelor’s degree (80%), and only 20% had a postgraduate degree. A total of 40% of the dietitians were employed in government hospitals, and 39% worked at other health-related facilities. More than half of the sample (56%) had less than five years of professional experience. Regarding self-reported environmental knowledge perceptions, 55% rated their own knowledge as high, but 58% rated their clients’ knowledge as low. The importance of sustainable diets was perceived by 77% of the dietitians; however, the reported frequency of delivering environmental information to clients was ‘when needed’ by 33% of the dietitians and only 26% reported an ‘always’ frequency. Higher percentages reported the consideration of social aspects during nutrition consultations (86%) (Table 1). The descriptive characteristics of the study population according to KAPs and sustainable food consumption scores are presented in Supplementary Table S1.

Table 2 shows the dietitians’ factual knowledge regarding the environmental impacts of different foods and dietary patterns. In total, 65% correctly identified beef as contributing the highest carbon dioxide (CO_2_) emissions, but only 31% knew that beef requires the highest water use. In addition, 44% correctly recognized the Lacto-ovo-vegetarian diet with small amounts of fish/chicken as the most sustainable dietary pattern. For the identification of the environmental impact of different food items, 49% correctly identified plant-based foods as “low”, 36% identified red meat as “high”, 43% identified processed meat as “high”, 24% identified dairy products as “high”, and 43% identified ultra-processed foods as “high” (Table 2). The mean and standard deviation of the sum score was 11 ± 4 (minimum 0, maximum 18), where 11% scored 0–6, 54% scored 7–12, and only 35% scored 13–18. Out of the eight questions, 61% correctly answered 0–3 questions, 27% correctly answered 4–5 questions, and only 12% correctly answered 6–8 questions. These findings show a limited level of factual knowledge of food sustainability. The standardized Cronbach’s alpha value for the factual knowledge domain was 0.662330, indicating questionable internal consistency.

Table 3 demonstrates the dietitians’ perceived knowledge regarding a few sustainability-related concepts. A total of 48% of the dietitians reported “I have a clear understanding of the term” for “local food”. For the “food sustainability” and “environmental impact” concepts, 40% and 47% of the dietitians selected “I have heard the term and know what it means”, respectively, indicating that these are relatively well-understood concepts. On the other hand, the concepts of “ecological footprint” and “carbon footprint” were not familiar, as 49% and 53% of the dietitians selected “no, I have not heard the term”, respectively (Table 3). Out of all the dietitians, 52% disagreed that healthy diets and sustainable diets mean the same, while 19% agreed. The mean and standard deviation of the sum score was 17 ± 6 (minimum 2, maximum 32), where 13% scored 2–10, 46% scored 11–18, 31% scored 19–25, and only 11% scored 26–32. These findings show a gap in perceived knowledge of food sustainability. The Cronbach’s alpha value for the eight statements within the perceived knowledge domain was 0.828412, indicating good internal consistency.

Table 4 shows the dietitians’ attitudes about the perceived importance of sustainability-related concepts. Dietitians rated the following contributors to a sustainable diet as “extremely important”: “reducing food waste” (77%), “easy-to-follow diet” (52%), “diet rich in vegetables and fruits” (50%), “with plenty of fresh products” (48%), “affordable foods” (47%) and “locally grown products” (31%). Out of the remaining items, the highest ratings of “very important” and “extremely important” were given for “no additives” (70%), ”organic food production” (66%), “reducing consumption of red meat and processed meat” (62%), “increasing the consumption of plant proteins” (61%), “respectful of biodiversity” (57%), and “diet with traditional foods from own culture” (55%). However, some contributors to a sustainable diet were ranked at lower levels of importance; for example, “consuming fish produced by aquaponics” (38%) and “reducing consumption of packaged water” (29%) were rated as “moderately important” (Table 4). The mean and standard deviation of the sum score were 47 ± 10 (minimum 0, maximum 68), where 2% scored 0–25, 13% scored 26–40, 69% scored 41–55, and only 16% scored 56–68. Overall, these findings indicate favorable attitudes toward the importance of sustainable dietary practices. The Cronbach’s alpha value for the 17 statements within the attitude domain was 0.862213, indicating good internal consistency.

Table 5 shows dietitians’ practices and readiness to engage in sustainability-related personal behaviors. A higher level of engagement (“I’m doing this confidently most of the time”) was reported for “reduce food waste” (70%), “consume plenty of vegetables and fruits” (49%), “consume plenty of fresh products” (46%), “choose locally grown products” (38%), “consume traditional foods from your own culture” (38%), and “reduce consumption of red meat and processed meat” (35%). A moderate level of engagement (“I have started to do this some of the time”) was reported for “increase consumption of plant proteins” (39%), “choose food with few ingredients” (38%), and “choose food with no additives” (38%). A lower level of engagement (“I’m thinking about this, but I need more information”) was reported for “choose low environmental impact food” (38%) and “choose food respectful of biodiversity” (38%), suggesting unfamiliarity with or low acceptance of this behavior. Lastly, a pattern of hindered implementation was observed for “choose organic food,” “reduce consumption of packaged water,” and “choose food with less packaging”; 32%, 30%, and 20% of dietitians, respectively, selected the “like to do this, but other things are stopping me” option (Table 5).

Regarding the dietitians’ willingness to pay more for sustainably produced food and beverages, 37% selected “somewhat willing” (37%), 32% selected “undecided” (32%), and only 10% reported being “willing”, which indicates the influence of economic consideration in sustainable food purchasing. The overall mean and standard deviation of the sum score are 39 ± 10 (minimum 2, maximum 64), where 2% scored 0–15, 19% scored 16–30, 52% scored 31–45, and 29% scored 46–64, suggesting overall relatively favorable practices for several healthy and sustainable dietary personal behaviors (Table 5). The Cronbach’s Alpha value for the 16 statements within the personal practices item was 0.821457, indicating good internal consistency.

Table 6 demonstrates dietitians’ dietary adherence to a study-specific index of food consumption related to food sustainability. Large percentages of respondents reported no consumption of meat alternatives (91%), non-dairy milk (77%) and processed meat (76%). Overall, low red meat consumption was reported, where the weekly frequencies of consumption were 1–2 (61%) followed by no consumption (19%). For eggs, dairy products, and white meat, 3–4 servings per week was selected by 38%, 34%, and 38% of respondents, respectively. Lower daily fruit and vegetable consumption was also observed, at 10% and 26%, respectively. Similarly, a moderate consumption of plant protein sourced from beans, lentils, chickpeas, fava beans, and nuts/seeds was observed, where 60% and 47% of respondents, respectively, reported a frequency of 1–2 servings per week. The mean and standard deviation of the sum score were 21 ± 4 (minimum 12, maximum 32), where 7% scored 12–15, 35% scored 16–20, 43% scored 21–25, and only 15% scored 26–32. These results reflect a frequent reliance on animal-based foods, a moderate consumption of fruits, vegetables, legumes, and nuts, and an extremely low incorporation of plant-based alternatives (Table 6). The Cronbach’s alpha value for the food consumption domain was 0.507904, indicating a low internal consistency.

Supplementary Table S2 demonstrates the Spearman correlation between each of the KAPs and sustainability dietary practices. Poor positive associations were seen for overall knowledge and practices (r_s_ = 0.21, *p* = 0.0003) and with food consumption (r_s_ = 0.24, *p* = 0.0001). A poor positive association was shown between attitudes and food consumption (r_s_ = 0.14, *p* = 0.0420). Practice shows fair correlations with food consumption (r_s_ = 0.45, *p* < 0.0001) and attitudes (r_s_ = 0.35, *p* < 0.0001) (Supplementary Table S2).

## 4. Discussion

The study aimed to investigate the knowledge, attitudes, practices, and dietary adherence to food sustainability among clinical dietitians in Saudi Arabia. One of the main findings was that the data showed a discrepancy between perceived and factual knowledge, where dietitians reported a slightly higher confidence in their own perceived knowledge, while they had a limited level of factual knowledge about food sustainability; nevertheless, in general, they had favorable attitudes toward food sustainability, with a limited incorporation of several personal sustainable food behaviors, in particular environmental sustainability, in their daily lives. Furthermore, food sustainability was not fully captured in all food consumption behaviors, where dietitians’ reported dietary adherence reflected frequent animal-based food consumption and only moderate consumption of fruits, vegetables, legumes, and nuts.

The current data showed that a slight majority of dietitians were confident in their perceived knowledge; however, a substantial variability in objective and factual food sustainability knowledge was reported. We saw an overall higher recognition of beef as a significant source of greenhouse gas emissions and plant-based foods as having a low environmental impact, but a large uncertainty in the recognition of water-intensive foods and in the most sustainable dietary patterns. This pattern of variability in knowledge has been reported among European dietitians, where they correctly identified the environmental impact of (1) vegetables, fruits, legumes, whole grains, and vegetable oils as low, (2) dairy, dairy alternatives, eggs, sugar, and fish as moderate, and (3) meat as high; however, meat alternatives were incorrectly considered as having a moderate instead of a low impact [22]. Furthermore, Saudi Arabian dietitians showed a higher familiarity with health-related aspects of sustainability compared to economic and social perspectives. This pattern of limited knowledge in economic and social sustainability was reported in previous studies [22,24,28]. Similarly, a gap in familiarity with environmental sustainability was documented [20], including for some complex environmental sustainability concepts, such as “farmland protection” [24] and “water footprint” [34]. Collectively, this lack of knowledge has been pointed out within dietetic practice in many countries [20,21,22,25,26], consistent with the reported lack of structured sustainability education [20,22]. Because a lack of knowledge has been a major barrier to the effective integration of sustainability into dietary guidance and practice [20,22,38], efforts are needed to mitigate this gap in knowledge.

Among Saudi dietitians, the overall theme of perceived importance (i.e., attitudes) toward food sustainability showed a higher importance placed on health-related components of sustainable diet compared to environment-specific components. This could reflect cultural variabilities in defining the importance of certain components of food sustainability. For example, we can compare our study with a similar study from a Spanish study of students and health professionals in the health sciences. Across the two studies, Saudi Arabian and Spanish respondents considered these factors very important at the following rates, respectively: importance of zero waste (77% vs. 58%), containing fresh products (48% vs. 63%), produced locally (31% vs. 62%), respectful of biodiversity (17% vs. 62%), and low environmental impact (21% vs. 60%) [34]. Future studies are needed to explore the region-specific importance of sustainability components within food habits and culture.

Moreover, Saudi Arabian dietitians reported a relatively higher engagement overall in sustainable personal food behaviors aligned with traditional health-related nutrition recommendations, such as the consumption of fruits, vegetables, and fresh foods and reducing food waste, while also reporting a lower adoption of sustainability considerations beyond human health principles, such as choosing food with a low environmental impact, reducing packaging, and choosing aquaponics-produced fish. An important factor to bear in mind while interpreting this finding is that the adoption of environmental-related behaviors might require advanced knowledge and skills with respect to the implementation of sustainable food purchases, such as knowledge of a product’s environmental footprint [22,38]. Regarding the additional cost of sustainably produced foods, only a small number of Saudi Arabian dietitians expressed a willingness to pay more, and the underlying reasons were not investigated in the current study. It is worth noting that studies from Saudi Arabia showed that local and traditional foods are an important factor in shaping dietary patterns [39], and knowledge and attitudes about food sustainability are linked with dietary choices [40], which might potentially explain the reasons for the unwillingness to pay more. For an international comparison, Irazusta-Garmendia et al. showed that Spanish students and professionals in health sciences had the highest favorable attitudes toward sustainable food production and purchasing sustainable foods; nevertheless, paying more for sustainable foods had a lower rate of agreement, which indicated that cost might be a barrier in their population [34]. These findings are consistent with the literature showing that cost is considered one of the barriers to adopting a sustainable diet, especially when environmentally sustainable food options are more expensive than their conventional alternatives [20,26,27].

The results showed an overall relatively low adherence to the study-specific index of food consumption related to food sustainability among Saudi Arabian dietitians, with more reliance on red meat and animal products and limited consumption of plant-based food on a daily basis, suggesting that dietary sustainability recommendations may not have been fully incorporated into their own dietary practices. A lack of full dietary adherence to a sustainable diet was previously reported among Spanish students and professionals in health sciences, where they reported a frequent intake of fruit and vegetables, moderate intake of dairy products, and a limited intake of red meat; however, approximately one-third of them reported an intake of processed meat at least 1–5 times per week [34]. It is worth noting that adherence to the index of self-reported food consumption related to food sustainability across cultural contexts might be limited due to structural barriers for sustainable food options, such as a lack of food access and affordability [20,26,27]. Although the observed pattern between attitudes and behaviors in the current study indicated a possible gap between these domains, this is in agreement with the literature, in which attitudes are not always represented by corresponding behaviors [41,42] because social and contextual factors, such as affordability, availability, and social norms, may limit the translation of attitudes into behaviors [43,44,45]. Furthermore, dietitians and personal dietary behaviors should not casually imply dietetic professional competence.

Despite growing interest in food systems and sustainability in dietetics, there have been insufficiencies in incorporating sustainable food system-related competency standards within dietetic training standards [46,47]. Higgins et al. evaluated international dietetic education and training content in North America, Europe, Asia, Africa, and Oceania in 2021 and showed that 70% of 23 standards documents included sustainable food systems or related concepts, and more than half of these used broad, cross-cutting language (i.e., sustainable and food systems) rather than granular specific terms (i.e., biodiversity, equity and food security) [46]. Similarly, Wegener et al. assessed the characteristics of incorporating sustainable food systems into accredited nutrition and dietetics programs in the U.K, Australia, and Canada via content analysis of university course descriptions between 2021 and 2022 and found inconsistent and limited integration, often limited to individual units within a course rather than as dedicated, independent courses, where more sustainability content was included in nutrition programs than dietetics programs [47]. Furthermore, the level of cognitive complexity in sustainable food system-related competency standards was at lower-order cognitive skills such as “understand and apply,” which indicated that dietitians were not expected to assess, appraise, or assist in sustainable food systems and sustainable dietary patterns’ advancement [46]. This is a huge limitation because convincing people to shift toward sustainable healthy diets is not just a matter of implementing an increase in plant-based consumption and a decrease in animal consumption; it requires trade-offs and careful consideration between food security, food safety, and environmental, social, and economic sustainability [48,49], while also mitigating nutrient deficiencies, as reliance on plant-based diets has reportedly been associated with an increased risk of deficiencies in nutrients such as vitamin B12, calcium, iron, and zinc in adults [13,50]. Dietitians should be qualified and equipped to provide balanced, sustainable dietary recommendations while preventing such nutrient deficiencies in their dietetics practices.

Therefore, it is required that nutrition and dietetics professionals apply systems thinking for problem-solving and leadership under conditions of uncertainty; these characteristics could provide insights for reforming curriculum design, training, and nutrition and dietetics practices [26], alongside regional-specific learning outcomes, equipping dietitians to positively and significantly contribute to sustainable food systems and diets [14,19,46,47]. In fact, it was pointed out that dietetics professionals’ contributions across socioecological systems via positions in clinics, public health, consumer demands, food service, policy, and food supply [51] could potentially achieve a cumulative high social impact in supporting sustainable food systems [26].

This study has a few strengths and limitations. The study examined a topic that is seldom investigated in the Middle Eastern regions. The target population is of special interest for facilitating the promotion of sustainable dietary patterns among the Saudi population, as they have a critical role in modifying dietary behaviors via the dietary recommendations targeted through their professional dietetic practice or community messages. The study findings shed some light on dietitians’ perceptions of sustainability, which is measured in order to modify educational curricula and professional training programs. However, the study has some limitations that should be acknowledged. First, the study has a small sample size, with a lack of an a priori sample-size calculation needed for statistical power analysis. Despite the lack of, an accurate national estimate of dietitians in Saudi Arabia with sex distribution, the female predominance in the study’s sample (80%) is generally in agreement with previous studies among dietitians in Saudi Arabia (74.4%) [52], Arab countries (83.1% [53] and 88.2% [54]), the U.S (95.3% [55] and 96% [56]), and Ireland (98%) [28,57]. Nevertheless, this study aimed to investigate current KAPs and adherence to a study-specific index of food consumption related to food sustainability rather than formulating generalizable conclusions for all dietitians in Saudi Arabia. This was not the aim, as indicated by the non-probability sampling approaches of convenience and snowball sampling and the observed imbalance in sex distribution.

Anonymous data were collected through two different platforms; therefore, the possibility of duplication from the two platforms cannot be eliminated. Nevertheless, only 11 (5.0%) responses of the total analytical sample of 221 were obtained through the Google Form, limiting the possibility of highly influencing the overall findings. Because of that, selection bias in the current explanatory analysis cannot be ruled out. Moreover, the use of self-reported data could introduce reporting bias and social desirability bias given dietitians’ professional expectations, in particular, with respect to food consumption. Because KAPs and food consumption were examined simultaneously, a common-method bias may have influenced the associations between these domains. Due to the overlap between some of the KPAs and food consumption in a few items, it is worth acknowledging that this overlap might have influenced the reported correlations between these domains; thus, caution has to be taken when interpreting these findings, and future studies might benefit from using distinct measures to accurately capture the correlations between these domains. Another limitation was the lack of stratified analyses, limiting our ability to observe subgroup-specific patterns; thus, future studies are warranted with larger sample sizes, and these should consider conducting stratified analyses. Although the survey that was used was adapted from previous studies [32,34], the final Arabic survey used in the current study has only been validated by experts in the field for face and content validity. Cronbach’s alpha was calculated to assess the internal consistency (i.e., reliability), with no rigorous or comprehensive psychometric assessment of validity and reliability. For the food consumption domain, the Cronbach’s alpha value indicated a relatively low internal consistency; this could reflect the multidimensional characteristics of food consumption, so the internal consistency needs to be interpreted with caution, as it does not merely indicate poor validity of the food consumption index. Nevertheless, future studies should be conducted to develop validated psychometric instruments for assessing sustainability KAPs and competencies in clinical applications and professional sustainability-focused practice for dietitians.

## 5. Conclusions

The current study captured the knowledge, attitudes, practices, and dietary adherence to food sustainability among Saudi Arabian dietitians. It was found that there was a discrepancy between perceived and factual knowledge; more than half of the dietitians rated their perceived knowledge as high, but limited levels of factual knowledge were seen. Also, an overall pattern suggested that dietitians had relatively higher favorable attitudes toward food sustainability and practiced several health-focused sustainable personal behaviors. Nevertheless, gaps were identified in environmental sustainability attitudes and practices, and these gaps did not consistently represent adherence to the study-specific food sustainability index, where food consumption personal behaviors were characterized by a frequent consumption of animal-based foods and moderate consumption of fruits. These findings have critical implications due to the increase in global attention to sustainable and healthy diets for mitigating the negative impact of food systems on the environment, the burden of non-communicable diseases, and the pivotal role of dietitians in influencing public dietary practices toward more sustainable food practices. Therefore, improving dietitians’ competencies for effective contributions toward more sustainable food systems should be a priority. As a result, the current dietetic education system might benefit from incorporating food sustainability content into the nutrition curricula of all food and dietetic programs in Saudi Arabia to mitigate the limited exposure to sustainability-related content and competencies. These should involve more hands-on experience, critical thinking, and higher cognitive skills rather than a passive didactic education style, building upon the previous studies in this area [58,59]. Furthermore, providing mandatory continuous professional education programs on food sustainability could help bridge the gap among current dietitians in the workforce.

Future studies could explore the characteristics of holistic sustainability training and continuous education hours directed toward current dietitians in the workforce and evaluate their effect on improving knowledge, skills, and dietetic competencies. Changes at the health-related governmental organization level can help the actual incorporation of food sustainability by providing evidence-based resources on sustainability principles for dietitians and practical resources for clients to help incorporate these principles into counseling sessions and community initiatives, and during food purchases. Furthermore, until the new sustainability dietetic education curriculum is fully endorsed, nationwide longitudinal studies are greatly needed to track the improvements in adherence to sustainable dietary practices among dietitians and the extent of the incorporation of food sustainability principles in their dietetics practice. Our results call for future studies to investigate the determinants of sustainable diet adherence among dietitians, including barriers and cultural influences. Future studies are warranted with the implementation of objective assessment tools that comprise comprehensive sustainability measures to improve the validity of results and enhance international comparisons. Additionally, the incorporation of qualitative assessments of the situation would yield a deeper understanding of the current situation by identifying barriers and facilitators, assessing the training and education needed, and indicating the methods of delivery for these skills to promote sustainable food practices with dietetic practice and education to help in guiding the smooth integration of a robust sustainability framework into dietetic practice within Saudi Arabia.

## Figures and Tables

**Table 1:** Descriptive characteristics of the study population (N = 221).

Variable	N (%)
Sex	
Male	45 (20)
Female	176 (80)
Marital status	
Single	126 (57)
Married	95 (43)
Region	
Central	72 (33)
Western	73 (33)
Eastern	22 (10)
Northern	21 (10)
Southern	33 (15)
Education level	
Bachelor’s	177 (80)
Postgraduate	44 (20)
Monthly income (SAR)	
<5000	25 (11)
5000–9999	55 (25)
10,000–13,999	43 (20)
14,000–19,999	47 (21)
≥20,000	51 (23)
Health facility type	
Government hospital	89 (40)
Private hospital	24 (11)
Private clinic	19 (9)
Training center	3 (2)
Other	86 (39)
Years of experience	
<5 years	123 (56)
5–10 years	53 (24)
>10 years	45 (20)
Self-rated environmental knowledge	
High	121 (55)
Moderate	82 (37)
Low	9 (5)
Don’t know	9 (5)
Clients’ environmental knowledge	
High	17 (8)
Moderate	57 (26)
Low	129 (58)
Don’t know	18 (9)
Importance of sustainable diet	
Important	170 (77)
Not important	19 (9)
Don’t know	32 (15)
Providing environmental information	
Always	58 (26)
Often	57 (26)
When needed	71 (33)
Never	35 (16)
Consider social aspects	
Yes	189 (86)
No	21 (10)
Don’t know	11 (5)

Abbreviation: SAR; Saudi Arabian Riyals. Data are presented as frequencies (N) and percentages.

**Table 2:** Dietitians’ factual knowledge regarding the environmental impacts of different foods and dietary patterns (N = 221).

Variable	Category	N (%)
Food with the highest CO_2_ emissions	Vegetables	58 (26)
	Chicken	10 (5)
	Fish	6 (3)
	**Beef**	144 (65)
	Don’t know	3 (1)
Food with the highest water use	Grains	93 (42)
	**Beef**	69 (31)
	Watermelon	39 (18)
	Lettuce	19 (9)
	Don’t know	1 (0)
The most sustainable diet	Lacto-ovo-vegetarian diet with moderate amounts of red meat	75 (34)
	**Lacto-ovo-vegetarian diet with small amounts of fish/chicken**	97 (44)
	Lacto-ovo-vegetarian diet (no red meat at all)	19 (9)
	Vegan diet (no animal protein at all)	25 (11)
	Don’t know	5 (2)
Environmental impact of plant foods	Don’t know	12 (5)
	**Low**	108 (49)
	Medium	50 (23)
	High	51 (23)
Environmental impact of red meat	Don’t know	16 (7)
	Low	41 (19)
	Medium	85 (38)
	**High**	79 (36)
Environmental impact of processed meat	Don’t know	27 (12)
	Low	80 (36)
	Medium	20 (9)
	**High**	94 (43)
Environmental impact of dairy products	Don’t know	16 (7)
	Low	32 (14)
	Medium	120 (54)
	**High**	53 (24)
Environmental impact of ultra-processed foods	Don’t know	30 (14)
	Low	69 (31)
	Medium	28 (13)
	**High**	94 (43)

Abbreviation: CO_2_; carbon dioxide. Data are presented as frequencies (N) and percentages (%). Bold values represent the correct answer for each question.

**Table 3:** Dietitians perceived knowledge regarding several sustainability-related concepts (N = 221).

Do You Know the Following Concepts?	No, I Have Not Heard the Term	I Have Heard the Term, but I Don’t Know What It Means	I Have a Vague Understanding of the Term	I Have Heard the Term and Know What It Means	I Have a Clear Understanding of the Term
Ecological footprint	109 (49)	42 (19)	30 (14)	27 (12)	13 (6)
Carbon footprint	117 (53)	38 (17)	26 (12)	26 (12)	14 (6)
Food sustainability	22 (10)	27 (12)	56 (25)	89 (40)	27 (12)
Environmental impact	17 (8)	14 (6)	43 (19)	104 (47)	43 (19)
Biodiversity	23 (10)	31 (14)	51 (23)	75 (34)	41 (19)
Local food	5 (2)	6 (3)	12 (5)	93 (42)	105 (48)
Greenhouse gas emissions	25 (11)	28 (13)	54 (24)	71 (32)	43 (19)
	Strongly agree	Agree	Neither agree nor disagree	Disagree	Strongly disagree
Do you believe that healthy diets and sustainable diets mean the same?	5 (2)	37 (17)	65 (29)	90 (41)	24 (11)

Data are presented as frequencies (N) and percentages (%).

**Table 4:** Dietitians’ attitudes regarding sustainability-related concepts (N = 221).

To What Extent Do You Consider That Each of the Following Contributes to a Sustainable Diet?	Not at All Important	Slightly Important	Moderately Important	Very Important	Extremely Important
Low environmental impact	12 (6)	30 (14)	66 (30)	67 (31)	46 (21)
Respectful of biodiversity	9 (4)	17 (8)	69 (32)	90 (41)	36 (17)
No additives	4 (2)	18 (9)	45 (21)	84 (38)	70 (32)
Fewer ingredients	14 (6)	43 (20)	76 (35)	59 (27)	29 (14)
Organic food production	9 (4)	21 (10)	46 (21)	83 (38)	62 (28)
Diet with plenty of fresh products	7 (3)	9 (5)	10 (5)	89 (41)	106 (48)
Diet rich in vegetables and fruits	5 (3)	5 (3)	18 (9)	84 (38)	109 (50)
Diet with traditional foods from own culture	13 (6)	23 (11)	64 (29)	70 (32)	51 (23)
Locally grown products	9 (4)	20 (9)	34 (15)	89 (41)	69 (31)
Affordable foods	5 (3)	9 (5)	27 (13)	76 (35)	104 (47)
Easy-to-follow diet	5 (3)	8 (4)	14 (6)	79 (36)	115 (52)
Choosing foods with less packaging	20 (9)	32 (15)	53 (24)	61 (28)	55 (25)
Reducing consumption of red meat (e.g., beef, lamb, mutton) and processed meat (e.g., sausages, hot dogs, nuggets)	8 (4)	27 (13)	49 (22)	57 (26)	80 (36)
Increasing consumption of plant proteins (e.g., beans, nuts, tofu, chickpeas, lentils)	6 (3)	21 (10)	60 (27)	85 (39)	49 (22)
Consuming fish produced by aquaponics	29 (13)	42 (19)	83 (38)	40 (18)	27 (12)
Reducing consumption of packaged water	22 (10)	25 (12)	63 (29)	62 (28)	49 (22)
Reducing food waste	4 (2)	3 (2)	5 (3)	39 (18)	170 (77)

Data are presented as frequencies (N) and percentages (%).

**Table 5:** Dietitians’ practices regarding a few sustainability-related personal behaviors (N = 221).

For Each Behavior Listed Below, Select a Statement That Most Applies to You.	I’m Not Interested in Doing This at the Moment	I’m Thinking About This, but I Need More Information	I Would Like to Do This, But Other Things Are Stopping Me	I Have Started to Do This Some of the Time	I’m Doing This Confidently Most of the Time
Choose low environmental impact food	34 (15)	83 (38)	54 (25)	39 (18)	11 (5)
Choose food respectful of biodiversity	30 (14)	84 (38)	42 (19)	48 (22)	17 (8)
Choose food with no additives	8 (4)	17 (8)	49 (22)	83 (38)	64 (29)
Choose food with few ingredients	29 (13)	28 (13)	41 (19)	83 (38)	40 (18)
Choose organic food	30 (14)	11 (5)	70 (32)	61 (28)	49 (22)
Consume plenty of fresh products	7 (3)	7 (3)	25 (11)	82 (37)	100 (46)
Consume plenty of vegetables and fruits	2 (1)	6 (3)	27 (12)	77 (35)	109 (49)
Consume traditional foods from your own culture	22 (10)	16 (7)	32 (15)	68 (31)	83 (38)
Choose locally grown products	12 (5)	18 (8)	32 (15)	74 (33)	85 (38)
Choose food with less packaging	45 (20)	39 (18)	44 (20)	54 (24)	39 (18)
Reduce consumption of red meat (e.g., beef, lamb, mutton) and processed meat (e.g., sausages, hot dogs, nuggets)	27 (12)	21 (10)	39 (18)	57 (26)	77 (35)
Increase consumption of plant proteins (e.g., beans, nuts, tofu, chickpeas, lentils)	27 (12)	14 (6)	50 (23)	87 (39)	43 (19)
Choose fish produced by aquaponics	76 (34)	38 (17)	51 (23)	38 (17)	18 (8)
Reduce consumption of packaged water	59 (27)	22 (10)	67 (30)	34 (15)	39 (18)
Reduce food waste	2 (1)	8 (4)	15 (7)	42 (19)	154 (70)
	Unwilling	Somewhat unwilling	Undecided	Somewhat willing	Willing
To what extent are you willing to pay more money for food and drinks that are produced in a sustainable way?	26 (12)	19 (9)	71 (32)	82 (37)	23 (10)

Data are presented as frequencies (N) and percentages (%).

**Table 6:** Dietitians’ dietary adherence to the study-specific food sustainability index based on self-reported food consumption (N = 221).

How Often Do You Consume Each of These Foods in a Typical Week?	0 Times	1–2 Times	3–4 Times	5–6 Times	7+ Times
Dairy (e.g., milk, cheese, yogurt, labneh)	5 (2)	45 (20)	75 (34)	56 (25)	40 (18)
White meat (e.g., chicken, turkey)	4 (2)	18 (8)	84 (38)	77 (35)	38 (17)
Nuts/seeds	17 (8)	103 (47)	64 (29)	30 (14)	7 (3)
Red meat (e.g., beef, lamb, camel meat)	41 (19)	134 (61)	34 (15)	10 (5)	2 (1)
Eggs	13 (6)	46 (21)	85 (38)	50 (23)	27 (12)
Processed meat (e.g., sausages, hot dogs, nuggets, beef bacon)	167 (76)	53 (24)	1 (0)	0 (0)	0 (0)
Beans, lentils, chickpeas, fava beans	18 (8)	133 (60)	53 (24)	12 (5)	5 (2)
Fish	58 (26)	133 (60)	29 (13)	1 (0)	0 (0)
Meat alternatives (e.g., tofu, soya products, plant-based sausages/burgers)	201 (91)	17 (8)	3 (1)	0 (0)	0 (0)
Non-dairy milk (e.g., soy, almond, coconut, oat)	170 (77)	36 (16)	10 (5)	5 (2)	0 (0)
Fruits	7 (3)	70 (32)	85 (38)	37 (17)	22 (10)
Vegetables (not including legumes and pulses)	5 (2)	32 (14)	65 (29)	62 (28)	57 (26)

Data are presented as frequencies (N) and percentages (%).

## Data Availability

The datasets generated and/or analyzed during the current study are available from the corresponding author upon reasonable request.

## Supplementary Materials

The following supporting information can be downloaded at: https://www.mdpi.com/article/10.3390/nu18182966/s1, Supplementary Table S1. Descriptive characteristics of the study population by KAP levels and sustainable food consumption (N = 221). Supplementary Table S2. Correlation between knowledge, attitudes, practices, and food consumption (N = 221).

## Abbreviations

The following abbreviations are used in this manuscript:

KAPs	Knowledge, Attitude, and Practices
