# Supplementary material for: Food Sustainability Among Dietitians in Saudi Arabia: Assessment of Knowledge, Attitudes, Practices, and Food Consumption
Source: Nutrients. 2026 Sep 10;18(18):2966. doi: 10.3390/nu18182966 (PMC13610294; doi:10.3390/nu18182966)
Supplement: Supplementary file 1 [file nutrients-18-02966-s001.zip › nutrients-4516770-supplementary.pdf]

Supplementary Table S1. Descriptive characteristics of the study population by KAP levels and sustainable food consumption (N=221)

Variable	N (%)	Factual knowledge	Perceived knowledge	Overall knowledge	Attitudes	Practices	Food consumption
		Median (Q1 - Q3)	Median (Q1 - Q3)	Mean ± SD	Median (Q1 - Q3)	Median (Q1 - Q3)	Median (Q1 - Q3)
**Gender**							
Male	45 (20)	11 (9–15)	19 (15–23)	0.16 ± 0.82	46 (42–51)	42 (36–48)	20 (18–24)
Female	176 (80)	10 (8–14)	17 (13–21)	−0.04 ± 0.78	48 (43–53)	39.5 (32–46)	21 (19–24)
**Marital status**							
Single	126 (57)	11 (9–14)	17.5 (14–21)	0.03 ± 0.78	47.5 (42–51)	38 (32–45)	21 (18–24)
Married	95 (43)	10 (8–14)	18 (13–22)	−0.04 ± 0.81	48 (43–54)	42 (36–49)	22 (20–25)
**Region**							
Central	72 (33)	11 (8.5–14)	18 (14.5–21.5)	0.05 ± 0.80	48 (44–54)	40.5 (32–48)	21 (19–24)
Western	73 (33)	9 (7–12)	16 (13–21)	−0.21 ± 0.82	49 (45–53)	39 (32–44)	21 (18–24)
Eastern	22 (10)	10 (9–11)	16 (13–19)	−0.17 ± 0.65	45 (41–51)	37 (32–46)	22 (18–24)
Northern	21 (10)	12 (9–15)	19 (17–24)	0.34 ± 0.79	47 (39–50)	42 (36–46)	23 (20–24)
Southern	33 (15)	12 (9–15)	19 (17–22)	0.25 ± 0.67	46 (41–53)	40 (33–46)	20 (18–24)
**Education level**							
Bachelor's	177 (80)	11 (9–14)	17 (13–21)	−0.04 ± 0.79	47 (42–52)	40 (32–46)	21 (18–24)
Postgraduate	44 (20)	10.5 (8–14.5)	18 (15–23)	0.14 ± 0.81	50 (46.5–57)	42.5 (36.5–48.5)	22.5 (21–25)
**Monthly income (SAR)**							
<5,000	25 (11)	11 (9–13)	18 (16–23)	0.03 ± 0.86	48 (42–53)	43 (30–46)	21 (18–23)
5,000–9,999	55 (25)	11 (9–13)	16 (13–20)	0.02 ± 0.64	45 (42–53)	39 (32–45)	21 (18–24)
10,000–13,999	43 (20)	10 (8–14)	17 (13–20)	−0.11 ± 0.83	48 (42–52)	40 (32–44)	21 (18–23)
14,000–19,999	47 (21)	11 (8–15)	19 (14–23)	0.07 ± 0.89	49 (44–54)	41 (36–50)	20 (18–24)
≥20,000	51 (23)	10 (8–14)	18 (13–22)	−0.01 ± 0.81	48 (44–53)	38 (32–49)	21 (20–25)
**Health facility type**							
Government Hospital	89 (40)	11 (9–14)	17 (13–21)	−0.08 ± 0.84	48 (43–53)	40 (35–48)	21 (19–23)
Private Hospital	24 (11)	11 (8–12.5)	16 (13–20.5)	−0.15 ± 0.65	45 (41.5–49)	38.5 (33.5–43)	22.5 (18–25)
Private Clinic	19 (9)	9 (8–11)	21 (16–26)	0.15 ± 0.71	49 (45–54)	45 (41–50)	23 (20–27)
Training Center	3 (2)	12 (1–13)	17 (2–19)	0.26 ± 1.61	40 (0–48)	38 (2–40)	21 (12–21)
Other	86 (39)	11 (9–15)	18 (15–22)	0.12 ± 0.75	47.5 (42–54)	38.5 (30–46)	21 (18–24)
**Years of experience**							
<5 years	123 (56)	11 (9–14)	18 (14–22)	0.06 ± 0.74	47 (42–52)	38 (30–45)	21 (18–23)
5–10 years	53 (24)	10 (8–13)	17 (13–19)	−0.15 ± 0.86	49 (44–54)	40 (36–46)	21 (19–25)
>10 years	45 (20)	11 (9–14)	18 (13–22)	0.01 ± 0.84	48 (45–54)	42 (36–50)	22 (20–25)
**Self-rated environmental knowledge**							
High	121 (55)	11 (9–14)	18 (15–22)	0.15 ± 0.82	48 (44–53)	42 (34–48)	21 (19–24)
Moderate	82 (37)	10 (8–13)	17 (13–20)	−0.14 ± 0.74	47 (42–53)	39 (32–46)	21 (18–24)
Low	9 (5)	12 (10–13)	14 (12–16)	−0.07 ± 0.28	47 (44–50)	29 (27–37)	20 (20–24)
Don't know	9 (5)	9 (5–11)	12 (7–19)	−0.66 ± 0.76	41 (38–55)	31 (21–40)	19 (17–21)
**Clients' environmental knowledge**							
High	17 (8)	11 (8–15)	18 (15–21)	0.01 ± 1.03	52 (44–59)	46 (39–50)	21 (17–23)
Moderate	57 (26)	10 (8–14)	19 (15–23)	0.10 ± 0.84	47 (42–54)	42 (34–48)	21 (18–26)
Low	129 (58)	11 (9–14)	17 (13–21)	0.003 ± 0.755	48 (44–52)	38 (32–46)	21 (19–24)

Don’t know	18 (9)	9 (7–10)	18 (11–19)	−0.33 ± 0.63	47.5 (41–54)	37.5 (32–40)	21 (17–22)
**Importance of sustainable diet**							
Important	170 (77)	11 (9–14)	18 (14–22)	0.07 ± 0.77	48 (44–53)	40.5 (34–47)	21 (18–24)
Not important	19 (9)	12 (9–16)	17 (15–21)	0.18 ± 0.62	43 (39–54)	33 (29–47)	22 (20–24)
Don’t know	32 (15)	9 (6.5–12)	15 (10.5–18)	−0.45 ± 0.90	48 (43–51)	36.5 (30.5–44)	21 (18.5–22)
**Providing environmental information**							
Always	58 (26)	11 (9–14)	19 (16–23)	0.22 ± 0.84	48 (44–52)	42.5 (38–50)	23 (19–26)
Often	57 (26)	11 (9–15)	18 (16–21)	0.15 ± 0.70	48 (42–54)	43 (33–48)	21 (19–23)
When needed	71 (33)	10 (8–13)	16 (13–21)	−0.11 ± 0.75	48 (43–53)	38 (30–45)	21 (18–25)
Never	35 (16)	10 (7–13)	15 (9–19)	−0.39 ± 0.80	47 (40–53)	34 (28–40)	20 (17–21)
**Consider social aspects**							
Yes	189 (86)	11 (9–14)	18 (13–22)	0.05 ± 0.78	48 (44–53)	40 (33–47)	21 (19–24)
No	21 (10)	9 (8–10)	15 (13–18)	−0.33 ± 0.80	48 (42–55)	38 (33–43)	22 (20–25)
Don’t know	11 (5)	12 (7–15)	15 (10–19)	−0.22 ± 0.94	42 (4–50)	30 (21–33)	20 (15–21)

Abbreviation: SAR; Saudi Arabian Riyals

Data are presented as frequencies (N) and percentages (%), mean ± standard deviation (SD), or median (first quartile (Q1), third quartile (Q3))

Supplementary Table S2. Correlation between knowledge, attitudes, practices, and food consumption (N=221)

Variables	Factual knowledge	Perceived knowledge	Overall knowledge	Attitudes	Practices	Food consumption
**Factual knowledge**	1.000	0.242 (**0.0003**)	0.749 (**<0.0001**)	−0.035 (0.6005)	0.023 (0.7307)	0.029 (0.6665)
**Perceived knowledge**	0.242 (**0.0003**)	1.000	0.797 (**<0.0001**)	0.165 (**0.0140**)	0.349 (**<0.0001**)	0.315 (**<0.0001**)
**Overall knowledge**	0.749 (**<0.0001**)	0.797 (**<0.0001**)	1.000	0.067 (0.3183)	0.239 (**0.0003**)	0.235 (**0.0004**)
**Attitudes**	−0.035 (0.6005)	0.165 (**0.0140**)	0.067 (0.3183)	1.000	0.351 (**<0.0001**)	0.137 (**0.0420**)
**Practices**	0.023 (0.7307)	0.349 (**<0.0001**)	0.239 (**0.0003**)	0.351 (**<0.0001**)	1.000	0.454 (**<0.0001**)
**Food consumption**	0.029 (0.6665)	0.315 (**<0.0001**)	0.235 (**0.0001**)	0.137 (**0.0420**)	0.454 (**<0.0001**)	1.000

Spearman correlation coefficients are presented.

Bold values indicate statistical significance ($P < 0.05$).
